# Supplementary material for: Runx1 Messenger RNA Delivered by Polyplex Nanomicelles Alleviate Spinal Disc Hydration Loss in a Rat Disc Degeneration Model
Source: Int J Mol Sci. 2022 Jan 5;23(1):565. doi: 10.3390/ijms23010565 (PMC8745749; doi:10.3390/ijms23010565)
Supplement: Supplementary file 1 [file ijms-23-00565-s001.zip › ijms-1491308-supplementary.pdf]

Supplementary Information

1. Supporting materials and methods

1.1. Synthesis of PEG-PAsp(DET) Block Copolymer and Nuclear Magnetic Resonance (NMR) Analysis

The PEG-PAsp(DET) block copolymer was synthesized as previously reported with slightly modification [1], based on an aminolysis of benzyl groups in the side chain of poly(β-benzyl L-aspartate) (PBLA) segment of PEG-PBLA block copolymer to generate N-substituted polyaspartamides (Figure 1A) bearing 2 repeating units of aminoethylene in the side chain. Briefly, aminolysis of PEG-PBLA to incorporate repeating aminoethylene units as the side chain was performed in dry DMF at 40 °C with diethylenetriamine (DET) for 24 h in the presence of a molar excess (50 equiv relative to benzyl groups) of aminoethylene donor (Figure S1A). We synthesized PEG (M.W. = 12,000)-poly{*N*-[*N′*-(2-aminoethyl)-2-aminoethyl]aspartamide}, possessing 2 repeating aminoethylene units, abbreviated as PEG-PAsp(DET). The degree of substitution of benzyl group with DET was determined by ^1H NMR analysis (400 MHz, D_2O) to be approximately 64 % (Figure S1B).

1.2. Construction of Vector for In Vitro Transcription (IVT) and Preparation of mRNA

The IVT vector used for production of *Luc2* mRNA was sub-cloned from pDNAs encoding photinus pyralis luciferase (pGL4; Promega Corporation) and insert the cDNA fragment into pSP73 vector (Promega Corporation) under the control of T7 promoter containing 120 bps chemically synthesized poly(d(A/T) fragments at the downstream of the cDNA region [3]. Then, the vectors were linearized with BsmBI, blunted with T4 DNA polymerase, purified with gel electrophoresis, and served as templates for IVT using the mMESSAGE mMACHINE T7 Ultra Kit (Ambion, Invitrogen, Carlsbad, CA) to generate mRNA. The mRNAs encoding Runx1 was similarly constructed from the vectors carrying the human *Runx1* ORF sequence (Runx1-FL), which was gift from Prof. Chung [2]. Prior to the experiments, all transcribed mRNAs were purified by RNeasy Mini kit (Qiagen) and analyzed for size and purity with the Agilent RNA 6000 Nano Assay on a BioAnalyzer 2100 (Agilent Technologies) (Figure S2B).

1.3. Preparation of Polyplex Nanomicelles and Physicochemical Properties Characterization

To prepare the mRNA polyplex nanomicelles, PEGylated polyaspartamides and mRNA were separately dissolved in HEPES buffer, and mixed at a volume ratio of 1:2. The concentration of mRNA was set to 666 ng/μL and that of PEGylated polyaspartamides were adjusted to obtain a residual molar ratio of amino groups in polymers to phosphate in mRNA (N/P ratio) of 3, and adjusted the mRNA quantity to 4 μg in a 6 μL total transfection volume. A 6 μL mRNA polyplex nanomicelles were diluted to 10-fold volume by ddH_2O for DLS (ZS90, Malvern, UK) and TEM (JEM-2100, JEOL, Japan) measurements. In DLS, 20 μL polyplex nanomicelles were

measured. In TEM, 3 μL polyplex nanomicelles were counter stained with uranyl acetate, and dipped onto TEM copper grid.

1.4. Cell Culture, Endocytosis of mRNA Loaded Polyplex Nanomicelles

The BMSCs were withdrew and mixed from the femora and tibias collected from two SD rats using a standard protocol and cultured in Dulbecco's modified Eagle medium (DMEM, Invitrogen, Carlsbad, CA) containing 10% fetal bovine serum (FBS, Thermo Scientific Hyclone, Rockford, IL), 100 IU/mL penicillin and 100 IU/mL streptomycin. The BMSCs were passaged and cells of passage 3 through 5 were used. To detect endocytosis of mRNA loaded polyplex nanomicelle, 1×10^5 BMSCs were seeded on 3.5-cm culture dish, supplemented with red fluorescent protein contained early endosome detection agent (CellLight™ Early Endosomes-RFP, Cat. C10587, Thermo Fisher Scientific Inc.) and medium for overnight culture. The Luc2 mRNA in 300 ng/μl was labeled with fluorescein using Label IT® Nucleic Acid Labeling Kit (Cat. MIR3225, Mirus Bio. LLC.) and electrostatically self-assembled with PEG-PAsp(DET) block copolymer in N/P ration of 3 for the preparation of Luc2 mRNA loaded polyplex nanomicelle, which is ready-to-use in the following 2-phontonic microscopy. Subsequently, the BMSCs were washed and re-supplemented with DAPI (Cat. D9542, Sigma-Aldrich) containing HBSS buffer in 5 μg/mL for 1h, followed with cell plasma membrane staining using orange fluorescent dye labeled detection agent for 20 min in compliance with manufacturer's instruction (Cell Navigator™ Cell Plasma Staining Kit, Cat. 22680, AAT Bioquest® Inc.). The BMSCs were washed, re-supplemented 2 mL complete medium and added 50 μL Luc2 mRNA loaded polyplex nanomicelle for immediate 2-phonton microscopic observation. The images were captured and lasted to 1 h.

1.5. Runx1 mRNA Transfection and Western Blot Analysis

To detect the integrity of runx1 encoded by mRNA delivery, BMSCs were cultured in 1×10^5 cells in 6-well overnight, transfected by Lipofectamine 3000 (Cat. L3000015, Thermo Fisher Scientific) in compliance with the manufacturer's instruction. Cell's protein was extracted with RIPA lysis buffer (Merck millipore, Cat. 20-188) containing 0.2% phosphatase inhibitor cocktail (Sigma-Aldrich, Cat. P2850), phenylmethylsulfonyl fluoride (Sigma-Aldrich, Cat. 52332) at 4 °C for 1 h, 15,000 rpm centrifugation 15 min for protein quantification using Pierce™ BCA Protein Assay Kit (Thermo Scientific, Cat. 23225). Proteins (10 μg) were electrophoresed by SDS-polyacrylamide gel and transferred to a PVDF (poly (vinylidene fluoride)) (Pall corporation, Cat. BSP0861) membrane. The membrane was blocked in 5% fat-free milk in PBST (PBS with 0.05% Tween-20), followed by incubation overnight with the following primary antibodies diluted in PBST: Runx1 (D-2) (Santa Cruz, Cat. sc-365644) and GAPDH (Santa Cruz, Cat. sc-32333) (diluted to 1:1000). The primary antibodies were removed, and the membrane was washed extensively in PBST. Subsequent incubation with horseradish peroxidase-conjugated goat anti-mouse antibodies (1:20,000, Santa Cruz, Cat. sc-2005) was performed at room temperature for 2 h. The membrane was washed extensively in PBST to remove any excess secondary antibodies, and

the blot was visualized with enhanced chemiluminescence reagent (GE Healthcare, Cat. GERPN2209).

1.6. Histological Examination

Rat tails were dissected and vertebral bodies encompassed coccygeal disc co4-5 were removed, fixed with 4% paraformaldehyde (PFA) in PBS, decalcified in 0.5 M EDTA (pH 8.0) for 2 weeks, dissected from the sagittal direction to equal two pieces of vertebral body, embedded in paraffin and serial sectioned in 5 µm thickness. Sagittal serial sections in the mid-zone of vertebral body were prepared for H&E staining and immunohistochemical (IHC) staining by standard protocol. Briefly, slides were de-paraffined, washed, blocked, and immuno-stained with rabbit anti-rat type II collagen primary antibody (Cat. sc-28887, Santa Cruz, US) at 4 °C overnight, then stained with Alexa 488-conjugated goat anti-rabbit secondary antibody (Cat. ab150077, Abcam, US) at room temperature for 1 h, then subsequently counter stained with DAPI (Cat. H-1200-10, VECTASHIELD®, VECTOR Laboratories, Inc., USA) and observed by fluorescence microscope (ImageXpress Pico, Molecular Device, USA).

Supplementary Figures

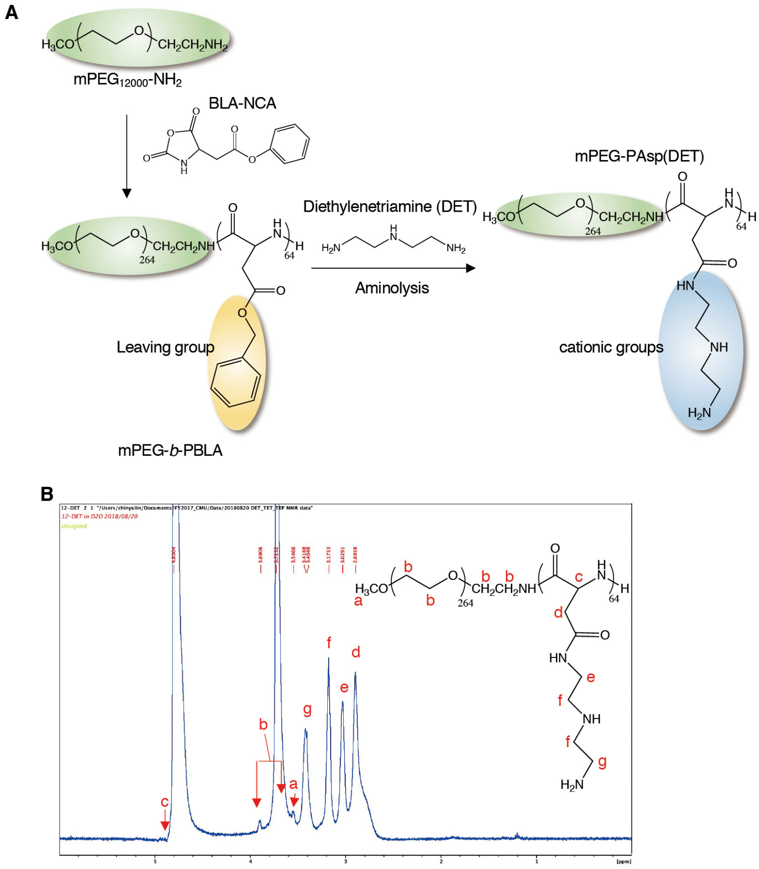

Figure S1. Synthesis of PEG-PAsp(DET) block copolymer and examination by NMR. **(A)** Synthesis of the block copolymer poly (benzyl l-aspartate)-*block*-poly(ethylene glycol) (PBLA-b-PEG) via ring-opening polymerization of β-benzyl l-aspartate-*N*-

carboxyanhydride (BLA–NCA) with α-methoxy- ϖ-aminopoly (ethylene glycol) (mPEG–NH_2) as a polymeration initiator, and followed with an aminolysis of benzyl groups in the side chain of PBLA segment of the PBLA-b-PEG block copolymer to generate N-substituted polyaspartamides. **(B)** ^{1}H-NMR spectrum for the PEG-PAsp(DET).

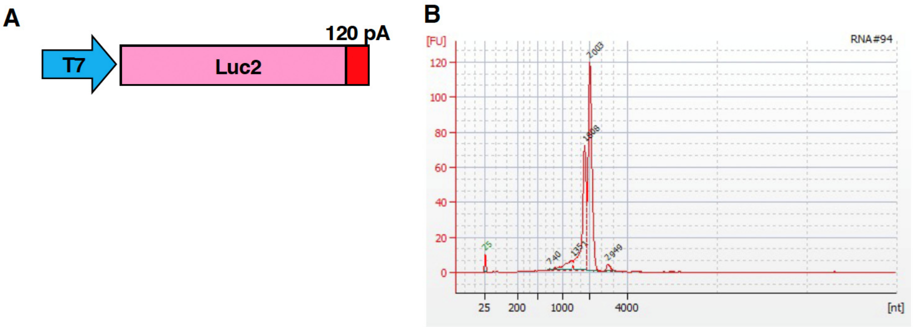

Figure S2. Vector for IVT to produce Luc2 mRNA and mRNA quality analysis. **(A)** Map of Luc2 mRNA IVT vector with 120pA tail in the end of Luc2 cDNA. **(B)** Bioanalysis data of Luc2 mRNA used in this study.

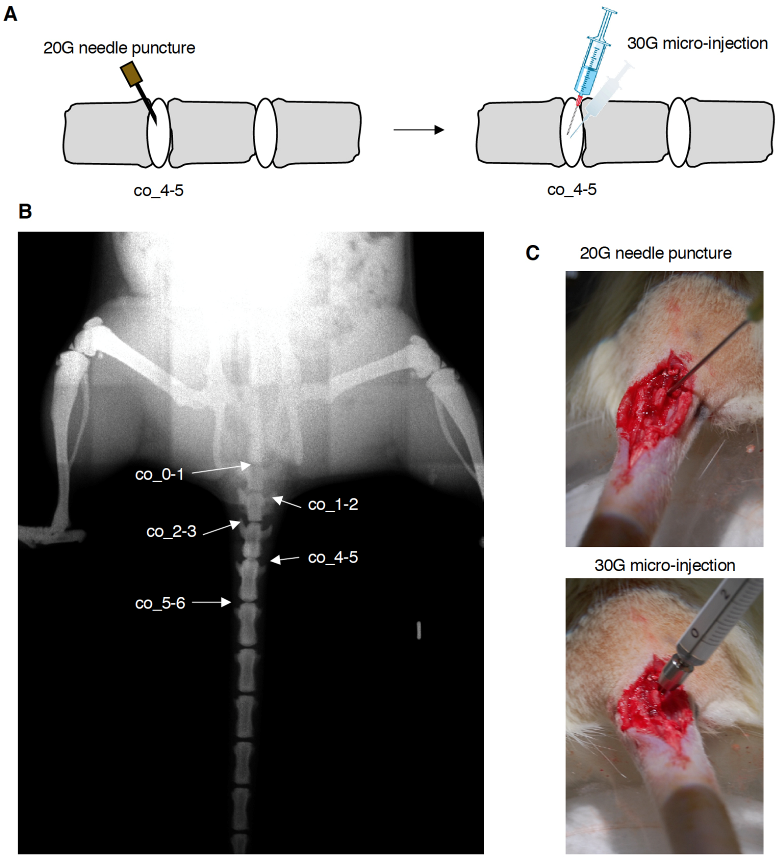

Figure S3. Establishment of coccygeal disc puncture in rat model and micro-injection. **(A)** Illustration shows the puncture in the co 4-5 disc with 20G needle, followed with micro-injection with 30G needle under stereo microscopy. **(B)** X-ray radiographic image shows the coccygeal discs. **(C)** Needle puncture in coccygeal disc in rat model.

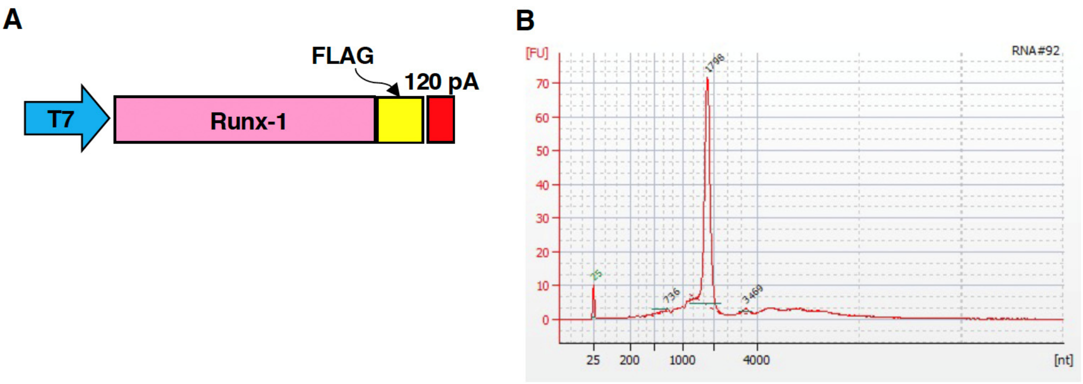

Figure S4. Vector for IVT to produce Runx1 mRNA and mRNA quality analysis. **(A)** Map of Runx1 mRNA IVT vector with 120pA tail in the end of Runx1 cDNA. **(B)** Bioanalysis data of Runx1 mRNA used in this study.

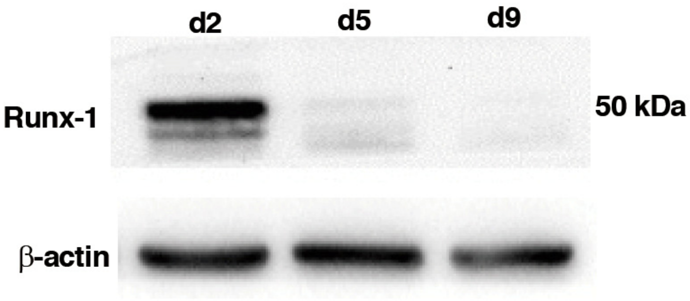

Figure S5. Western blot to examine the Runx1 transcription factor expression**.** Runx1 mRNA was transfected in BMSCs, and the cell lysate was collected for 1st Ab detection against Runx1 transcription factor and the β-actin housekeeping protein. Representative blotting data shows the abundant Runx1 transcription factor expression at dpt 2, demonstrated the integrity of Runx1 mRNA for protein translation. (N=3).

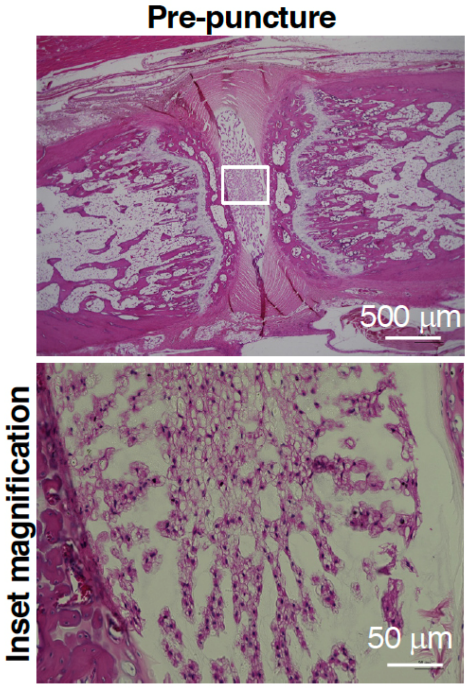

Figure S6. H&E staining of intact rat coccygeal disc. H&E staining shows the intact annular fibrous tissues and jelly-like materials in the disc core for comparison with puncture damaged disc.

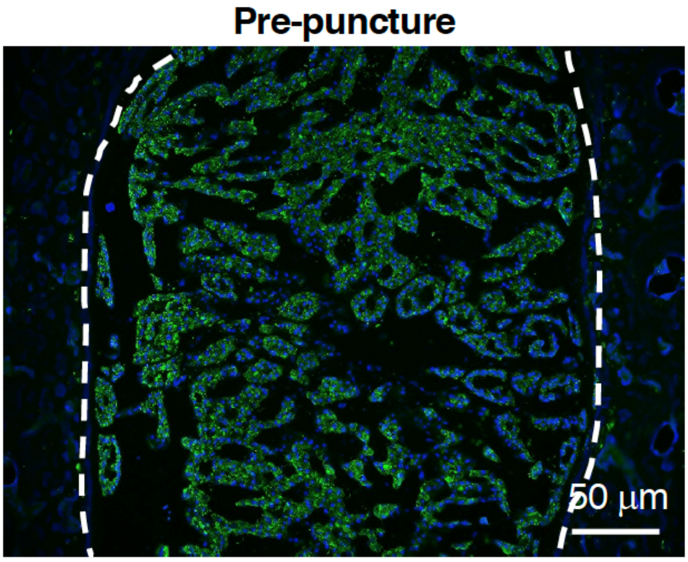

Figure S7. IHC staining of intact rat coccygeal disc detect the Collagen II. IHC stained with 1st Ab recognize the type II collagen and the 2nd Ab prelabeled with Alexa 488. For comparison with puncture damaged disc, the jelly-like materials demonstrated the abundant collagen II in the ECM composition in the intact disc core. Dashed line circled the jelly-like materials in the disc core.

References

1. Chan, L.Y.; Khung, Y.L.; Lin, C.-Y. Preparation of Messenger RNA Nanomicelles via Non-Cytotoxic PEG-Polyamine Nanocomplex for Intracerebroventicular Delivery: A Proof-of-Concept Study in Mouse Models. *Nanomaterials* **2019**, *9*, 67, https://doi.org/10.3390/nano9010067.
2. Aini, H.; Itaka, K.; Fujisawa, A.; Uchida, H.; Uchida, S.; Fukushima, S.; Kataoka, K.; Saito, T.; Chung, U.-I.; Ohba, S. Messenger RNA delivery of a cartilage-anabolic transcription factor as a disease-modifying strategy for osteoarthritis treatment. *Sci. Rep.* **2016**, *6*, srep18743, https://doi.org/10.1038/srep18743.
3. Holtkamp, S.; Kreiter, S.; Selmi, A.; Simon, P.; Koslowski, M.; Huber, C.; Türeci, O.; Sahin, U. Modification of antigen-encoding RNA increases stability, translational efficacy, and T-cell stimulatory capacity of dendritic cells. *Blood* **2006**, *108*, 4009–4017, https://doi.org/10.1182/blood-2006-04-015024
